# Tracheal Diverticulum in SARS-CoV-2 Patients on Non-Invasive Ventilation A not so “Spontaneous” Cause of Pneumomediastinum? An imaging Pictorial Presentation of Two Cases with Review of Literature

**DOI:** 10.15388/Amed.2021.28.2.19

**Published:** 2021-12-22

**Authors:** Veena Mariam Joseph, Donboklang Lynser, Iadarilang Tiewsoh, Kaustuv Dutta, Pranjal Phukan, Chhunthang Daniala

**Affiliations:** Department of Radiology and Imaging, NEIGRIHMS, North Eastern Indira Gandhi Regional Institute of Health and Medical Sciences, Mawdiangdiang, Shillong, Meghalaya, India; Department of Radiology and Imaging, NEIGRIHMS, North Eastern Indira Gandhi Regional Institute of Health and Medical Sciences, Mawdiangdiang, Shillong, Meghalaya, India; Department of Medicine, NEIGRIHMS, North Eastern Indira Gandhi Regional Institute of Health and Medical Sciences, Mawdiangdiang, Shillong, Meghalaya, India; Department of Anaesthesiology, NEIGRIHMS, North Eastern Indira Gandhi Regional Institute of Health and Medical Sciences, Mawdiangdiang, Shillong, Meghalaya, India; Department of Radiology and Imaging, NEIGRIHMS, North Eastern Indira Gandhi Regional Institute of Health and Medical Sciences, Mawdiangdiang, Shillong, Meghalaya, India; Department of Radiology and Imaging, NEIGRIHMS, North Eastern Indira Gandhi Regional Institute of Health and Medical Sciences, Mawdiangdiang, Shillong, Meghalaya, India

**Keywords:** COVID-19, NIV, pneumomediastinum, SARS-CoV-2 pneumonia, tracheal diverticulum

## Abstract

Spontaneous pneumomediastinum is a rare but potentially life-threatening condition, the incidence of which has showed an increase in patients with SARS-CoV-2 pneumonia, especially when they are on positive pressure ventilation. None of the reported cases of covid related pneumomediastinum had an associated tracheal diverticulum. Also, to the best of our knowledge, tracheal diverticulum has not been reported in patients on NIV. We report 2 cases of COVID-19 pneumonia on NIV with pneumomediastinum, which also had associated tracheal diverticulum, one of which developed after NIV. Though the establishment of causality needs further research, early detection of a tracheal diverticulum, which might be a harbinger of pneumomediastinum, can be a timely alarm to prompt titration of the pressure settings and judicious use of NIV. The role of inverted grey scale CT images in mediastinal window is a simple, yet hardly utilised radiological tool to increase detection of ‘mediastinal air’, let it be free air or air within a diverticulum. Through this case report, we would like to highlight the role of conventional and inverted CT imaging of pneumomediastinum and tracheal diverticulum in general and in SARS-CoV-2 pneumonia in particular, and to call for more objective research to throw light on the plausible relationship between pneumomediastinum and tracheal diverticulum.

## Abstract (lt)

**SŪRS CoV-2 pacientų, kuriems taikoma neinvazinė plaučių ventiliacija, trachėjos divertikulas. Ne visai savaiminio pneumomediastinumo atvejis? Dviejų atvejų vaizdinis pristatymas ir literatūros apžvalga:**

**Santrauka. **Savaiminis pneumomediastinumas yra reta, bet pavojų gyvybei kelianti būklė, kuri dažniau pasitaiko SŪRS CoV-2 plaučių uždegimu sergantiems pacientams, ypač jei jiems taikoma teigiamo slėgio ventiliacija. Nė vienu su COVID-19 susijusiu pneumomediastinumo atveju nepasireiškė trachėjos divertikulas. Be to, mūsų žiniomis, trachėjos divertikulo nebuvo nustatyta pacientams, kuriems taikyta neinvazinė ventiliacija. Aprašome du COVID-19 plaučių uždegimo atvejus, kai buvo taikoma neinvazinė ventiliacija ir pasireiškė trachėjos divertikulas, vienu atveju jis pasireiškė taikius neinvazinę ventiliaciją. Nors galimas priežastinis ryšys turėtų būti geriau ištirtas, ankstyvas trachėjos divertikulo nustatymas gali pranašauti pneumomediastinumą ir būti įspėjamuoju ženklu, kad reikia titruoti slėgio nustatymus ir apgalvotai naudoti neinvazinę ventiliaciją. Atvirkšti tarpuplaučio KT vaizdai yra paprasta, bet retai naudojama radiologinė priemonė, kuri padeda aptikti orą tarpuplautyje, nepaisant to, ar tai laisvas oras, ar oras, esantis divertikule. Aprašydami šį atvejį, norime pabrėžti įprasto ar atvirkščio KT tyrimo vaidmenį nustatant pneumomediastinumą ir trachėjos divertikulą apskritai ir SŪRS CoV-2 plaučių uždegimą konkrečiai, ir paraginti atlikti objektyvesnį galimo pneumomediastinumo ir trachėjos divertikulo ryšio tyrimą.

**Raktažodžiai:** COVID-19, neinvazinė ventiliacija, pneumomediastinumas, SŪRS CoV-2 plaučių uždegimas, trachėjos divertikulas

## Introduction

Since the beginning of the pandemic in December 2019, severe acute respiratory syndrome corona virus 2 (SARS-CoV-2) infection is nothing like its predecessors, in terms of persistence, genetic evolution and diverse clinical manifestation. Spontaneous pneumomediastinum is a rare but potentially life-threatening condition. There has been an increase in incidence of spontaneous pneumomediastinum in patients with SARS-CoV 2 pneumonia, especially when they are on positive pressure ventilation. 

The incidence of pneumomediastinum in COVID-19 patients have been reported to be around 6% in a study conducted by Loffi et al. in 102 cases of COVID-19 pneumonia [1]. The exact pathophysiology is not understood but the most suggested mechanism is the Macklin effect [2]. This effect states that the inciting event leading to spontaneous pneumomediastinum is alveolar injury, which in turn causes interstitial emphysema and air tracking along the peribronchovascular interstitium. The cause of alveolar injury can be barotrauma, cytokine storm leading to diffuse alveolar injury or direct infection of pneumocytes with the virus [3]. Most of the patients with severe pneumonia complicated with hypoxemic respiratory failure goes on to require some form of positive pressure ventilatory support. Non-invasive ventilation (NIV) is initiated in hypoxic patients, who are conscious, cooperative and able to protect the airway. It has been used as a bridging therapy to evade invasive ventilation in hypoxemic respiratory failure.

To the best of our knowledge none of the reported cases of covid related pneumomediastinum had an associated tracheal diverticulum. We report here imaging findings using conventional CT scan along with the not often used inverted CT protocol in 2 cases of COVID-19 pneumonia on NIV with pneumomediastinum, which also had associated tracheal diverticulum, one of which developed after NIV. 

## Case 1

**Figure 1.**
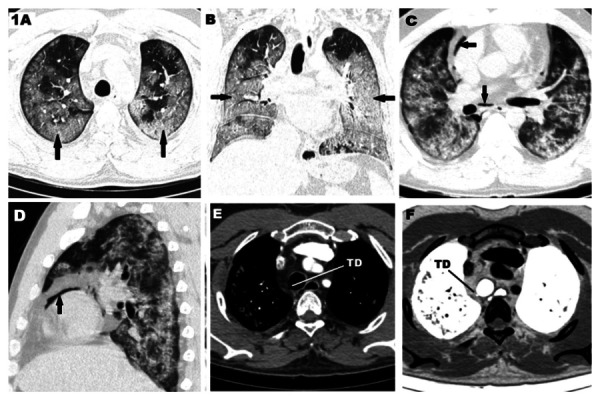
**CT scan of a 50-year-old male, RT- PCR positive SARS-CoV-2 patient showing. A. **Axial HRCT scan showing multifocal ground glassings in both lung fields typical of covid 19 lesions (arrows). **B.** Coronal HRCT scan showing extensive involvement in both lungs (arrows). **C&D.** Axial and sagittal CT scan showing pneumomediastinum (arrows) along with lung lesions aﬅer non-invasive ventilation. **E&F.** showing the tracheal diverticulum (TD) in axial mediastinal window and axial inverted CT images respectively.

50-year-old man presented with fever, cough and dyspnoea for 3 days. He was RT-PCR positive for SARS-CoV-2. CT thorax with thin sections was done which showed extensive confluent ground glassing with interlobular septal thickening diffusely involving bilateral lung with patchy area of sparing in bilateral apices (Fig. 1 A-B). There was also sparing of immediate subpleural lung in most areas. He had a protracted course in the hospital with persistent hypoxia requiring non-invasive ventilation for 7 days. A CT pulmonary angiogram was done to rule out pulmonary embolism in view of persistent sinus tachycardia. There was no evidence of pulmonary embolism. Lung involvement was similar to the initial study (Fig. 1 C-D). A small focal outpouching was noted from the right posterolateral wall of trachea at the level of D4 vertebra indicating tracheal diverticulum in both conventional and inverted CT imaging along with scattered foci of mediastinal air (Fig. 1 C-F). On reviewing the previously done images there was no evidence of tracheal diverticulum even on the thin cuts (1 mm).

He was weaned off the NIV by gradually decreasing the pressure support and oxygen concentration, until the saturation was maintained above 94% on room air.

## Case 2

**Figure 2.**
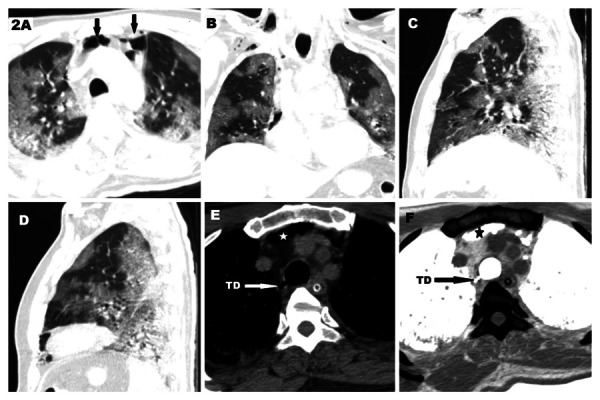
**CT scan of a 54-year-old male with severe Covid 19 pneumonia. A. **axial CT scan in lung window showing multiple peripheral ground glassing with pneumomediastinum (arrows) . **B–D.** Coronal, Sagital of right lung and Sagittal of left lung respectively showing peripheral ground glassings typical of covid 19 pneumonia. **E. **Axial CT scan in mediastinal window showing tracheal diverticulum (TD) and pneumomediastinum (star). **F.** Inverted CT image of the same showing better depiction of the diverticulum (TD).

54-year-old man presenting with cough, breathlessness and fever for 5 days. He was RT-PCR positive for SARS-CoV-2. He had severe clinical pneumonia and CT severity score of 21/25. He required non-invasive ventilation to maintain oxygen saturation above 94%, with ventilatory settings of pressure support of 8 cm H_2_O and a positive end expiratory pressure (PEEP) of 5 cm H_2_O. Total duration of NIV support was 11 days. CT thorax was obtained which revealed confluent peripheral ground glassing with a few areas of interlobular septal thickening in bilateral lungs, predominantly in the lower lobes (Fig. 2 A-D). The overall lung involvement was given a score of 21/25 (severe pneumonia). The CT also revealed pneumomediastinum in the retrosternal location of the anterior mediastinum and subcutaneous plane of neck (Fig. 2 E-F). A small focal outpouching was noted from the right posterolateral wall of trachea (Fig. 2 E). This was better appreciated after inverting the images in mediastinal window to allow better contrast between the mediastinal fat and air (Fig. 2 F).

He worsened clinically and finally succumbed to the disease on the 15^th^ day of admission.

## Discussion

The primary target organ of involvement of SARS-CoV-2 infection is the lung. Pulmonary manifestations include pneumonia with alveolar and interstitial infiltrates manifesting on imaging as bilateral ground glassing or consolidation predominantly involving the periphery and lower lobes. Other rare thoracic radiologic findings in these patients include pneumothorax, pneumomediastinum and pneumopericardium.

Tracheal diverticulum is one of the causes of paratracheal air. An acquired tracheal diverticulum is classically defined as a focal outpouching of the mucosa through the point of weakness in the tracheal wall, most often located in the lateral wall where the trachealis muscle inserts into the C shaped cartilaginous ring. The incidence of incidentally detected tracheal diverticula is up to 6% [4]. There have been isolated case reports in the past where pneumomediastinum and tracheal diverticula were incidentally detected [5-8]. Some of these cases of diverticula and pneumomediastinum were in patients after endotracheal intubation and ventilation [7]. It is interesting to note that despite the presence of tracheal diverticulum, there was no definite perforation of these areas on bronchoscopy [8].

In our cases of pneumomediastinum in COVID-19 pneumonia, both of the patients had diverticulum in the distal trachea. In one of them we had a prior CT scan at the time of admission which did not show any evidence of diverticulum. We were not able to demonstrate the peribronchial or perivascular air that has been described with the Macklin phenomenon [2]. Both our patients had received non-invasive ventilation during the course of hospital stay. There has been a reported case of an acquired tracheal diverticulum due to a malpositioned endotracheal tube abutting the carina with increased cuff pressure, possibly causing the diverticulum [9]. However, there is no reported case of tracheal diverticulum with NIV. We speculate that repeated coughing in in our patient coupled with the positive pressure ventilation from NIV could have led to the development of the diverticulum in the weakened tracheal wall.

The possibility of air leak from a tracheal diverticulum, which is a point of weakness of the airway needs to be considered. This will help in keeping a high index of suspicion for this finding in patients with COVID-19 and especially those on high positive airway pressures. Early recognition of this entity and alerting the physician regarding a possibility of incipient pneumomediastinum may help in preventing the same with a reduction in the pressure support or PEEP in the ventilatory setting if the situation permits it. We would also like to highlight the role of grey scale inversion of images to get an improved contrast between mediastinal fat and air and a resultant increased sensitivity in detecting both pneumomediastinum as well as tracheal diverticulum. And while inverted gray-scale imaging has been proved to be not superior to digital conventional X-ray for diagnosis of pneumomediastinum [10], yet the advantage of an inverted CT image over a conventional CT scan has not yet been explored. To the best of our knowledge there is dearth of literatures pertaining to the use of inverted CT imaging in chest radiology or elsewhere.

One of the limitations in our cases is that tracheo-bronchoscopic confirmation was not done and another limitation in one of our cases is that baseline scan to know the time of occurrence of the tracheal diverticulum was not available.

## Conclusion

Through our representation of two cases of severe COVID-19 pneumonia requiring NIV, which subsequently develop pneumomediastinum and incidental tracheal diverticulum, we would like to raise the suspicion of a logical association between these entities. However, more research on this is needed to establish causality. Early detection of a tracheal diverticulum, which might be a harbinger of pneumomediastinum, can be a timely alarm to prompt titration of the pressure settings and judicious use of NIV. Last but not the least, the role of inverted grey scale CT images in mediastinal window is a simple, yet hardly utilised tool to increase detection of ‘mediastinal air’, let it be free air or air within a diverticulum. 

## References

[ref1] Loffi M, Regazzoni V, Sergio P, Martinelli E, Stifani I, Quinzani F, Robba D, Cotugno A, Dede M, Danzi GB. Spontaneous pneumomediastinum in COVID-19 pneumonia. Monaldi Arch Chest Dis. 2020 Sep 29;90(4). doi: 10.4081/monaldi.2020.1399. PMID: . 32990690 10.4081/monaldi.2020.1399

[ref2] Marsico S, Del Carpio Bellido LA, Zuccarino F. Spontaneous Pneumomediastinum and Macklin Effect in COVID-19 Patients. Arch Bronconeumol. 2021 Jan;57 Suppl 1:67. doi: 10.1016/j.arbres.2020.07.030. Epub 2020 Aug 27. PMID: ; PMCID: . 10.1016/j.arbres.2020.07.030 PMC7451191 34629663

[ref3] Machiraju PK, Alex NM, Safinaaz, Baby NM. Pneumomediastinum in COVID-19: A series of three cases and review of literature. SAGE Open Med Case Rep. 2021 Apr 29;9:2050313X211011807. doi: 10.1177/2050313X211011807. PMID: ; PMCID: . 34017591 10.1177/2050313X211011807 PMC8114250

[ref4] Marina Pace, Annarita Dapoto, Alessandra Surace, Alessio Di Giacomo, et al. Tracheal diverticula: A retrospective analysis of patients referred for thoracic CT. Medicine(Balitimore) 2018 Sep;97(39):e12544. doi: 10.1097/MD.0000000000012544. PMID: 30278548 10.1097/MD.0000000000012544 PMC6181548

[ref5] Mazul-Sunko B, Zdenčar D, Kožul I, Spiček-Macan J, Stančić-Rokotov D. Pneumomediastinum related to distal tracheal diverticulum. Anaesthesia. 2013 Apr;68(4):432–3. doi: 10.1111/anae.12193. PMID: . 23488851 10.1111/anae.12193

[ref6] Cherrez-Ojeda I, Felix M, Vanegas E, Mata VL. Pneumomediastinum, Tracheal Diverticulum, and Probable Asthma: Coincidence or Possible Association? A Case Report. Am J Case Rep. 2018 Oct 25;19:1267–1271. 10.12659/AJCR.911413. PMID: ; PMCID: . 30356031 10.12659/AJCR.911413 PMC6213822

[ref7] Chakraborty A, Vaish R, Chatterjee A, Sable N, Chaukar D. Rare Presentation of Known Entity: A Case Report. A A Pract. 2020 Jul;14(9):e01262. doi: 10.1213/XAA.0000000000001262. PMID: . 32909716 10.1213/XAA.0000000000001262

[ref8] Varutti R, Rosa F, Zuccon U, Bassi F. The accidental discovery of a tracheal diverticulum. Intensive Care Med. 2019 Feb;45(2):259–260. 10.1007/s00134-018-5316-4. Epub 2018 Jul 24. PMID: . 30043274 10.1007/s00134-018-5316-4

[ref9] Sharma M, Bulathsinghala CP, Khan A, Surani SR. An Unusual Case of Iatrogenic Tracheal Diverticulum Found in a Mechanically Ventilated Patient: To Treat or Not to Treat. Cureus. 2019 Oct 15;11(10):e5911. doi: 10.7759/cureus.5911. PMID: ; PMCID: . 31788371 10.7759/cureus.5911 PMC6855996

[ref10] Musalar E, Ekinci S, Ünek O, Arş E, Eren HŞ, Gürses B, Aktaş C. Conventional vs invert-grayscale X-ray for diagnosis of pneumothorax in the emergency setting. Am J Emerg Med. 2017 Sep;35(9):1217–1221. doi: 10.1016/j.ajem.2017.03.031. Epub 2017 Mar 18. PMID: . 28343817 10.1016/j.ajem.2017.03.031

